# Silent, but not innocent: the emerging role of clonal hematopoiesis in predicting therapy-related myeloid neoplasms

**DOI:** 10.3389/fonc.2026.1943135

**Published:** 2026-09-30

**Authors:** Şerife Emre Ünsal

**Affiliations:** Department of Hematology, Faculty of Medicine, Erciyes University, Kayseri, Türkiye

**Keywords:** clonal hematopoiesis, DNA damage response, hematopoietic stem cell transplantation, therapy-related myeloid neoplasms, TP53 mutation

## Abstract

Therapy-related myeloid neoplasms (t-MNs), which develop following cytotoxic chemotherapy or radiotherapy, remain one of the most significant clinical challenges in modern hematology due to their aggressive clinical course and poor survival outcomes. The traditional paradigm regarded the development of t-MNs as a consequence of *de novo* genetic damage induced by anticancer therapies. However, recent data concerning genomics and clonal evolution have fundamentally transformed our understanding of the biological origins of the disease. Current evidence demonstrates that low-level, clinically silent hematopoietic clones are present in the peripheral blood or bone marrow years prior to disease onset in a substantial proportion of t-MN cases. The pathogenesis of t-MNs is therefore being reconceptualized, not merely as the emergence of *de novo* treatment-induced mutations, but rather as the expansion and progression to malignant transformation of pre-existing silent clones under selective pressure. Clonal hematopoiesis (CH) is a dynamic biological process characterized by the acquisition of a proliferative advantage by specific cell populations, resulting from somatic mutations accumulated by hematopoietic stem cells (HSCs) throughout life. A clinically defined subgroup of this process, including CH of indeterminate potential (CHIP) and clonal cytopenia of undetermined significance (CCUS), are defined as the presence of somatic mutations with a variant allele frequency (VAF) of ≥2% in genes associated with myeloid malignancies. Both are recognized as clinically meaningful pre-cancerous states, differentiated primarily by the presence of unexplained cytopenias in CCUS. The presence of CH by itself is insufficient to drive malignant transformation. The observation that only a small fraction of individuals harboring CHIP or CCUS ultimately develop myeloid neoplasia indicates that clonal progression is not solely dictated by the presence of a mutation, but rather by the biological characteristics of the clone, the specific mutation type, clone size, environmental stressors, and the bone marrow microenvironment. The fundamental paradigm has thus shifted from simply asking “which patients have CH?” to determining “which silent clones possess the potential to evolve into aggressive myeloid neoplasms in the future?” The concept of the “power of silent clones” provides a novel biological perspective in t-MN risk stratification, establishing a foundational framework for the future development of personalized surveillance, early intervention, and targeted preventative strategies. Deciphering CH enables precise risk stratification and helps intercept myeloid malignancies before clinical onset.

## 1 Introduction

Clonal hematopoiesis (CH) is an age-related physiological condition characterized by the acquisition of somatic mutations or copy number alterations that confer a selective proliferative advantage on hematopoietic stem cells (HSCs). The prevalence of CH increases markedly with advancing age. A more clinically relevant sub-classification of CH, known as clonal hematopoiesis of indeterminate potential (CHIP) and clonal cytopenia of undetermined significance (CCUS), is present in approximately 10% of individuals over 60. This is defined by the presence of a somatic mutation in a gene associated with myeloid malignancy (1).

Somatic cells continuously accumulate mutations throughout human life. The fifth edition of the World Health Organization Classification of Hematolymphoid Tumors and the International Consensus Classification formally describe both CHIP and CCUS as clinically significant pre-cancerous conditions. Both are characterized by the detection of one or more somatic mutations in genes associated with myeloid malignancies, with a variant allele frequency (VAF) of at least 2%, in the blood or bone marrow (2, 3). While CHIP is identified based on the absence of unexplained cytopenia, CCUS is distinguished by its presence.

Silent clones are defined as non-malignant, pre-leukemic hematopoietic clones without the overt morphological and clinical features of active myeloid neoplasia but that contain somatic mutations conferring a selective advantage. The most compelling evidence elucidating their role in the pathogenesis of therapy-related myeloid neoplasms has emerged from studies investigating mutations in TP53, PPM1D, TET2, DNMT3A, and spliceosome genes. In particular, mutations within DNA damage response genes confer a profound selective advantage under the evolutionary pressure exerted by cytotoxic therapies. HSCs harboring TP53 and PPM1D mutations acquire a distinct survival advantage by evading the apoptotic response to chemotherapy- or radiotherapy-induced DNA damage, ultimately driving their gradual expansion into dominant clonal populations.

Overall, only a small fraction of individuals with CHIP develop hematological malignancies during their lifetimes. Factors predictive of an increased risk of progression include a higher VAF, the presence of ≥2 mutations, and mutations in specific genes. Notably, these high-risk alterations involve spliceosome genes (such as SRSF2, SF3B1, and U2AF1), IDH2, JAK2 (particularly predisposing to myeloproliferative neoplasms), and TP53 (which is strongly associated with therapy-related neoplasms (t-MNs)) (4–7).

Three novel risk prediction models have recently been introduced to the literature for predicting the risk of myeloid neoplasm development in individuals diagnosed with CH and CCUS: CHRS, the “MN-predict” model, and the “Clonal Cytopenia Risk Score” (CCRS) (7–9). These seek to optimize clinical risk stratification and assessment processes using population-based data. The CHRS model incorporates the presence of CCUS, mutational patterns, patient age, red blood cell indices, and additional clinical factors as key parameters. In the corresponding study, individuals with a CHRS of ≥12.5 were classified into a high-risk cohort, demonstrating a five-year cumulative incidence of progression to a myeloid neoplasm of 24% (7). Another risk stratification tool, the MN-predict model, integrates genotypic, phenotypic, and biochemical data to estimate the annual probability of myeloid neoplasm transformation over a 15-year period (8). In addition to these established systems, recent research has introduced a novel prognostic model known as the CCRS. This model is based on three core parameters: the presence of an RNA splicing mutation, a platelet count of <100 × 10^9^/L, and the co-occurrence of two or more mutations (9). This clinically significant model has the potential to optimize individualized patient management, standardize clinical trials, and accelerate the development of novel, CCUS-specific therapies. However, the CHRS exhibits a higher discriminatory power, evidenced by a larger C-index, compared to the CCRS. Furthermore, owing to its robust predictive capacity, the CHRS is currently being employed to define clinical trial eligibility for CCUS trials (10).

Crucially, while these models exhibit significant clinical usefulness in the general population and *de novo* settings, it must be emphasized that none has been validated for risk prediction in the specific context of t-MNs. This represents a critical gap in current risk stratification paradigms, since the evolutionary dynamics of CH clones under cytotoxic selective pressure differ fundamentally from age-related clonal expansion.

t-MNs typically develop several years following exposure to radiotherapy or chemotherapy. Due to their aggressive clinical course and adverse survival outcomes, they represent one of the most formidable clinical challenges in modern hematology. Close monitoring of therapy-related CHIP, which can emerge following prior cytotoxic or radiation therapies, is highly important for predicting progression to high-risk clinical states. t-MNs exhibit a markedly more aggressive clinical trajectory than *de novo* malignancies. The median overall survival (OS) time post-diagnosis is a mere eight months, with a five-year survival rate of less than 10% (11). A previous study showed that one-year OS rates in t-MN ranged from 56% to 100% when stratified by patient gender, treatment type (allogeneic stem cell transplantation, chemotherapy), and specific subdiagnosis (12). Takahashi et al. demonstrated that clonal hematopoiesis mutations were already detectable in the peripheral blood prior to the initiation of primary cancer therapy in 74% of t-MN cases. In light of these data, research has shown that the presence of clonal hematopoiesis increases the risk of t-MN development four-fold under the selective pressure of cytotoxic stress (5).

There is currently no established standard-of-care for CHIP, and response rates to existing therapeutic modalities remain persistently low. It is therefore essential to identify high-risk patients for inclusion in novel clinical trials aimed at halting disease progression.

Elucidating the molecular mechanisms underlying clonal expansion and lineage differentiation will pave the way for strategies designed to mitigate adverse clinical risks while potentially harnessing any protective aspects of this biological process. The present review focuses on the CHIP and t-MN axis, with particular emphasis on the predictive power and clinical significance of silent clones in risk stratification.

## 2 Inflammation-driven clonal expansion in clonal hematopoiesis

During the aging process, somatic mutations steadily accumulate within the HSCs of numerous individuals, predisposing these to the development of CH. The pathogenesis of these conditions is driven by various mutational processes, together with the uncontrolled proliferation of pro-inflammatory myeloid lineages, such as macrophages and mast cells.

Chronic, low-grade inflammation associated with aging (inflammaging) impairs the function of normal HSCs in the bone marrow, reducing their self-renewal potential, while conferring a selective survival and growth advantage on mutant clones such as TET2 and DNMT3A (13) While normal stem cells are prone to exhaustion under inflammatory stress, these specific mutant clones exhibit resistance to stress and proliferate disproportionately. Increased interleukin (IL)-1β in aging bone marrow directly enhances the self-renewal capacity of TET2-deficient HSPCs. This cytokine upregulates the expression of genes (Erg, Meis1, and Egr1) that support cell maintenance and limit differentiation in TET2-mutant cells. In contrast, normal HSPCs undergo cellular exhaustion under chronic IL-1β exposure (14, 15). In normal HSCs under chronic IL-1β inflammation, the PU.1 transcription factor prevents abnormal growth (excessive expansion) by limiting protein synthesis and cell cycle activity (16, 17). However, TET2 mutations disrupt this suppressive function of PU.1 by causing aberrant methylation at PU.1 binding sequences. This causes TET2-mutant cells to expand uncontrollably by maintaining active protein synthesis and cell cycle progression, even under inflammatory stress (18). As a foundational study exploring the association between TET2-driven CHIP and atherosclerosis, the authors revealed that Tet2-mutant macrophages release increased amounts of IL-1β. Furthermore, they showed that inhibiting the NLRP3 inflammasome effectively neutralizes this atherogenic impact (14, 19).

TET2-deficient HSPCs gain a survival advantage by upregulating anti-apoptotic pathways in response to inflammatory cytokines in their environment. Following TNF exposure, the expression of pro-apoptotic genes (Tnfrsf1a, Tnfrsf1b, Fas, Casp3, and Casp8) is suppressed, while that of anti-apoptotic molecules (Bcl2, Birc2) is increased (20).

The concept of mutation-driven CH first appeared in the literature in 2012, when acquired TET2 mutations were linked to age-related skewing of X-chromosome inactivation (21).

*In vivo* models have demonstrated that Dnmt3a^R878H/+ mutant HSCs engrafted into aged recipient mice exhibit a significant long-term proliferative advantage compared to their wild-type counterparts. Furthermore, the aged bone marrow microenvironment has been shown to enhance the lymphoid progenitor capacity of these mutant cells, thereby facilitating a higher mutant B cell output.

Transcriptomic RNA sequencing analyses from aged murine models provide robust evidence that mutant HSC expansion is directly mediated by the TNF-α signaling pathway. The upregulation of tumor necrosis factor (TNF)-α observed within the aged bone marrow niche points to a pathological mechanism that closely mirrors the biology of DNMT3A-mutated human CH (22).

In addition to genetic alterations, the chronic inflammatory microenvironment is recognized as a critical determinant in the onset and progression of CH. Inflammatory cytokines not only foster the expansion of pre-existing mutant clones, but also shape clonal selection by directly influencing the self-renewal capacity, cell fate, and differentiation programs of HSCs. In particular, TET2-mutant HSCs acquire a selective advantage in the face of inflammatory stimuli, thus facilitating the eventual dominance of these mutant clones. Among these inflammatory mechanisms, TNF-α is one of the most extensively investigated mediators. Experimental ex vivo studies have revealed that while exogenous TNF-α exposure suppresses the proliferation and self-renewal capacity of wild-type HSCs, TET2-mutant HSCs exhibit profound resistance to this TNF-mediated suppression. With this selective survival advantage, these mutant cells preserve their self-renewal capabilities while markedly skewing their differentiation trajectory toward the lymphoid lineage (22).

Findings corroborated by single-cell transcriptomic analyses have revealed that TNF-α, a primary mediator of the chronic inflammatory microenvironment, exerts a receptor-specific dual effect in mutant CH. Following activation by inflammatory stimuli, the TNF-α/TNFR1 signaling axis enables mutant cells to evade apoptosis, thereby sustaining their proliferation and self-renewal capacity. Conversely, the TNF-α/TNFR2 signaling axis orchestrates the lineage commitment of HSCs toward the lymphoid, rather than the myeloid, lineage via chromatin modifications. These findings indicate that under inflammatory stress, the regulation of clonal burden and mature blood cell differentiation are governed by mutually independent molecular mechanisms (22).

The functionally divergent roles of TNF-α receptors have been further validated by means of genetic deletion models. TNFR1 deletion abrogates the competitive advantage and self-renewal capacity of mutant HSCs, triggering apoptosis without altering their lymphoid or myeloid differentiation trajectories. Conversely, while TNFR2 deletion preserves the competitive dominance of mutant cells, it disrupts the differentiation equilibrium. It suppresses lymphoid development and consequently drives the expansion of pro-inflammatory myeloid cells. Meanwhile, pan-TNF inhibitors, which achieve simultaneous inhibition of both receptors, suppress the competitive advantage of mutant cells, although they induce a complex phenotype characterized by marked, concurrent myeloid progenitor differentiation (22).

In addition to TNF-α, the IL-1 signaling pathway has emerged as a crucial therapeutic target in the context of CHIP. Research has demonstrated that receptor-specific Il1r1 gene deletion or pharmacological inhibition via anakinra, a Federal Drug Administration-approved IL-1 receptor antagonist, effectively suppresses IL-1β-mediated aberrant myeloid expansion, ameliorates splenomegaly, and significantly prolongs OS in both *in vivo* models and genetically engineered human hematopoietic stem cell models (1). These findings suggest that pharmacological targeting of the IL-1 signaling axis represents a promising early intervention strategy for abrogating TET2-driven myeloproliferation and averting subsequent clonal progression.

Recent studies increasingly emphasize the central role of inflammation in the pathogenesis of CH. In particular, TET2 mutations have been shown to confer a selective advantage upon HSCs within the inflammatory microenvironment, rendering them resistant to the suppressive effects of pro-inflammatory cytokines. This process establishes a positive feedback loop that promotes the expansion of mutant clones via the upregulation of IL-6 production (23–25). Furthermore, the translocation of microbial products into the systemic circulation, as a result of compromised intestinal barrier integrity, further amplifies IL-6 production. This, in turn, accelerates disease progression by enhancing the proliferation of TET2-mutant progenitor cells (26, 27).

Inhibition of the IL-6 signaling axis has been shown to significantly attenuate the cardiovascular risk associated with CH. At the same time, specific pro-inflammatory cytokines have been shown to selectively foster the expansion of DNMT3A-mutant clones (28, 29). Considered together, these findings indicate that the inflammatory cytokine network acts as more than just a microenvironmental driver of clonal expansion; it also plays a decisive role in the selective outcompetition of clones harboring distinct mutations. The therapeutic targeting of inflammatory pathways, particularly TNF-α, IL-1, and IL-6, therefore, represents a highly promising future strategy for suppressing specific CH subtypes and mitigating the risk of leukemic transformation.

## 3 General molecular drivers of clonal hematopoiesis

### 3.1 TET2 biology

TET2 is a fundamental epigenetic regulator that mediates active DNA demethylation by catalyzing the oxidation of 5-methylcytosine. It thus plays a critical role in orchestrating the self-renewal, proliferation, and differentiation processes of HSCs (30, 31). It governs gene expression by modulating methylation patterns across myriad genomic loci, including gene bodies, enhancer regions, and DNA methylation canyons (32, 33). Despite exhibiting biochemically antagonistic functions to DNMT3A, mutations in both genes yield a convergent phenotype characterized by enhanced stem/progenitor cell proliferation, impaired differentiation, and the subsequent development of CH (34, 35). This indicates that distinct epigenetic dysregulations can culminate in shared pathobiological outcomes. The prevailing consensus of opinion suggests that TET2, similarly to DNMT3A, epigenetically modulates gene expression to promote cellular differentiation, while simultaneously repressing aberrant self-renewal capacity (35).

The great majority of TET2 mutations identified in CH are loss-of-function alterations, representing one of the most frequently encountered genetic abnormalities in CHIP cohorts (36–39). *In vivo* murine models have shown that hematopoietic stem and progenitor cells (HSPCs) harboring a Tet2 deletion exhibit a markedly enhanced self-renewal capacity, thus conferring a selective growth advantage on the mutant clones (36–40). However, this genetic advantage becomes substantially more pronounced following interaction with the inflammatory microenvironment, thus accelerating disease pathogenesis. Indeed, elevated systemic levels of pro-inflammatory cytokines, especially IL-1β, IL-6, and TNF-α, have been demonstrated in both TET2-mutant individuals and murine models (23, 27, 41).

This inflammatory cytokine network, further amplified by secondary stressors such as intestinal microbiota alterations and atherosclerosis, exerts a robust selective pressure that favors the aberrant myeloid expansion of mutant cells (27, 42). In mechanistic terms, IL-6 and TNF-α promote cellular survival by conferring increased apoptosis resistance in TET2-mutant cells compared to wild-type HSCs. In contrast, the IL-1β signaling cascade directly stimulates long-term HSCs and myeloid-biased multipotent progenitors in the bone marrow and spleen, driving their proliferation. Under this inflammatory stress, rather than committing to terminal differentiation, Tet2-deleted stem cells preserve the stem cell pool by remaining quiescent in the G0/G1 phase of the cell cycle; they consequently skew their differentiation trajectory toward the myeloid lineage, predominantly generating pro-inflammatory MHCII^+^ macrophages (1, 20, 23).

Single-cell transcriptomic (scRNA-seq) and epigenetic profiling studies have elucidated the molecular mechanisms by which Tet2-deleted HSCs adapt to the inflammatory microenvironment. Mutant cells exposed to IL-1β-mediated inflammatory stress have been shown to become enriched for transcriptional programs associated with inflammation tolerance and self-renewal capacity, thereby upregulating genes that support cellular survival, such as Erg, Meis1, and Egr1 (1). From an epigenetic perspective, while wild-type stem cells undergo global DNA demethylation under inflammatory stress, Tet2-deleted cells resist this process, preserving a hypermethylated state. This hypermethylation is particularly pronounced at genomic loci regulating lymphoid and erythroid differentiation, as well as at the binding sites of key transcription factors governing terminal differentiation, such as PU.1, ELF4, CEBPB, and JUN. This epigenetic reprogramming ultimately precludes the terminal differentiation of mutant cells, thereby fueling their sustained proliferation and aberrant myeloid expansion (1).

### 3.2 DNMT3A mutations

DNMT3A is a fundamental epigenetic regulator that orchestrates *de novo* DNA methylation in HSCs, thus playing a critical role in determining cell fate, controlling self-renewal capacity, and sustaining cellular differentiation (43, 44).

*In vivo* murine models have shown that the loss of DNMT3A markedly enhances the self-renewal capacity of HSCs, while suppressing their differentiation, leading to the progressive accumulation of phenotypically normal, yet functionally defective stem cells (45–47). During this process, pronounced hypomethylation occurs, particularly at enhancer regions and DNA methylation canyons; secondary to this, the expression of genes involved in maintaining the stem cell phenotype, such as Hoxa9, Meis1, and Evi1, is upregulated (45). Analogous epigenetic alterations and upregulations in self-renewal-associated gene expression have been corroborated in DNMT3A-mutated human hematopoietic cells (48, 49). However, an isolated DNMT3A mutation is widely acknowledged to be insufficient to initiate malignant transformation. The progressive accumulation of additional genetic events is required for the development of acute myeloid leukemia (AML) and other hematological malignancies. Furthermore, the biological effects of DNMT3A in driving CH cannot be attributed to DNA methylation perturbations alone. Researchers have postulated that histone modifications, functional interactions with TET proteins, three-dimensional chromatin organization, and methylation-independent epigenetic mechanisms also contribute significantly to this process.

DNMT3A mutations rank among the most frequently identified genetic alterations in CH, accounting for approximately 50% of all cases (39, 50). In the context of CH, these mutations primarily manifest as heterozygous loss-of-function alterations and exhibit a broad distribution across the entire gene. In contrast to myeloid neoplasms, mutations in CH cases do not exhibit pronounced clustering at the R882 hotspot (51).

Nevertheless, the precise localization of the mutation is a crucial determinant of both residual enzymatic activity and biological behavior. The dominant-negative DNMT3A R882 variant drastically curtails functional enzyme activity to approximately 20% of wild-type levels, while in non-R882 heterozygous mutations, residual enzymatic activity is preserved at roughly 50% (52, 53). Researchers have proposed the existence of an inverse correlation between the decrease in residual DNMT3A activity and both the proliferation rate of mutant clones and the subsequent risk of AML development (52, 53). Establishing a risk stratification system based on mutation type and localization is therefore of paramount importance, particularly for the rigorous monitoring of high-risk individuals harboring the R882 mutation and for the development of prophylactic strategies aimed at averting AML transformation (52–54).

The biological implications of DNMT3A mutations extend beyond mere clonal expansion. In particular, DNMT3A-driven CH has been shown to confer a survival advantage in hematopoietic stem cell transplant recipients by promoting the generation of mutant T-lymphoid cells, thus contributing to the preservation of functional T-cell immunity in the elderly population (55, 56).

### 3.3 JAK2 V617F and clonal evolution

The JAK2 V617F mutation represents one of the pivotal driver mutations in CH, detected in approximately 3% of cases, and responsible for the constitutive activation of the JAK-STAT signaling pathway (57). The frequent manifestation of this mutation as the solitary genetic driver event in myeloproliferative neoplasms highlights its decisive role in HSC biology. Studies involving experimental murine models have shown that HSCs harboring the JAK2 V617F mutation acquire a modest competitive growth advantage compared to their wild-type counterparts (58, 59). However, comprehensive single-cell transcriptomic and functional analyses have revealed that the JAK2 V617F mutation actually reduces the long-term self-renewal capacity of HSCs, while conversely driving the proliferation and expansion of myeloid progenitor cells. This phenomenon not only promotes the expansion of differentiated cell populations, but also facilitates clonal evolution by establishing a biological milieu conducive to sustained clonal expansion and the progressive accumulation of additional somatic mutations (60).

## 4 Molecular drivers of therapy-related clonal hematopoiesis

Cytotoxic chemotherapy and radiotherapy regimens are capable of functioning as prominent environmental drivers, shaping the selection of CH clones. Notably, clones harboring mutations in genes associated with DNA damage response (DDR) pathways (TP53, PPM1D, and CHEK2) acquire a pronounced proliferative advantage and undergo marked clonal expansion following exposure to cytotoxic cancer therapies, outcompeting clones with other genetic alterations (4). Indeed, competitive clonal dynamic analyses within a cohort of 34 patients harboring concurrent DDR and non-DDR mutations demonstrated that DDR-mutant clones acquire a statistically significant selective growth advantage under cytotoxic stress compared to non-DDR-mutant clones residing within the same patient (4). Moreover, the evaluation of 34 additional cases that progressed from post-treatment CHIP to t-MN revealed that 59% of the t-MN cases already harbored at least one driver mutation during the CH stage, whereas 41% possessed two or more. Remarkably, TP53 mutations were identified in 44% of the t-MN cohort, and the presence of the mutation was confirmed to be detectable as early as the CHIP stage in 73% of these specific cases. Conversely, the complex karyotype abnormalities observed in 73% of the patients following t-MN development were notably absent during the antecedent CHIP phase (4).

### 4.1 DNA damage response pathways

TP53 and PPM1D are particularly prominent among the most frequently mutated DNA DDR genes in cases of CH, with PPM1D mutations being detected in approximately 3–4% of the cohort in one study (50). While early studies predominantly associated PPM1D alterations with non-hematological malignancies, contemporary data have revealed that these mutations are strongly correlated with prior exposure to cytotoxic therapies and the subsequent development of therapy-related acute myeloid leukemia (t-AML) (61–64).

It is important to remember that the evolutionary trajectories and progression rates to t-MN differ significantly between TP53 and PPM1D mutations. While TP53 mutations possess a robust intrinsic capacity to drive malignant transformation under cytotoxic stress, isolated PPM1D mutations appear insufficient to trigger t-MN development on their own. Recent data demonstrate that PPM1D-mutant clones typically require the acquisition of concurrent genetic events, such as TP53 co-mutations or complex cytogenetic aberrations, to progress fully to a myeloid neoplasm. Although PPM1D mutations are rapidly selected and enriched following genotoxic therapies, the risk of developing t-MN, whether these mutations are present in isolation or else co-occur with TP53 mutations, remains strikingly lower compared to TP53-mutant or double wild-type cases. A dynamic clonal competition unfolds between PPM1D and TP53 mutant clones during the progression toward malignancy. Under the selective pressure exerted by chemotherapy, PARP inhibitors (PSRPis), or radiopharmaceuticals, PPM1D mutations are selected early, driving clonal expansion and precipitating marked cytopenias. However, upon transitioning to the phase of malignant progression, PPM1D-mutant clones are superseded or entirely outcompeted by TP53-mutant clones, which possess a more aggressive and robust clonal advantage. Serial sampling analyses corroborate that, in the rare subset of patients who progress to t-MN, the PPM1D-mutant clone contracts, evidenced by a declining variant allele frequency (VAF) or becomes completely undetectable at the time of disease progression (65).

PPM1D is a phosphatase that functions as a negative regulator of the DNA damage response, suppressing the DDR signaling pathway by dephosphorylating various cellular targets, most notably p53 (61, 66). Mutations arising in this gene augment the stability of the protein, which leads to a further attenuation of DDR function (61, 66). Experimental studies have revealed that PPM1D-mutant cells acquire a selective advantage through a mechanism distinct from that of DNMT3A and TET2 mutations. By gaining a pronounced competitive superiority over wild-type cells due to their enhanced survival capacity, particularly following cytotoxic chemotherapy, these mutant clones can eventually achieve population dominance driven by the cumulative impact of recurrent treatment cycles. Furthermore, the resulting clonal expansion can remain stable over extended periods (61). During this dynamic process, shifts in the VAF are dictated by factors such as the initial mutation burden, the absolute number of hematopoietic cells, and the intensity of the therapy administered (61).

Similarly to TP53 mutations, researchers have postulated that PPM1D mutations bolster cellular survival under cytotoxic stress by enhancing apoptosis resistance, thereby conferring a marked selective advantage upon the mutant clones (67).

The precise mechanism by which PPM1D-mutant cells acquire a selective advantage remains to be fully elucidated. Although the fact that these mutations suppress chemotherapy- or radiotherapy-induced apoptosis is well established, it is still unclear how the inhibition of apoptosis directly contributes to mutation accumulation within HSCs. Furthermore, it is still uncertain whether this survival advantage is mediated exclusively through p53 dephosphorylation, or if other cellular targets within the DDR pathway also contribute to this process.

Regulated by TP53 expression, PPM1D functions as a negative regulator of p53 activity, thereby abrogating DNA damage-induced apoptosis (61, 66). The frameshift and nonsense mutations frequently observed in CH generate a truncated PPM1D protein with enhanced stability. This mutant protein subsequently dephosphorylates various DDR components, effectively inhibiting chemotherapy- and radiotherapy-induced, DDR-dependent apoptosis (61). In contrast to TP53 mutations, PPM1D mutations do not exhibit a strong correlation with pronounced genomic instability or poor prognosis, and they can be detected at low VAF levels, even in clinically healthy individuals. Nevertheless, despite their typically low clonal burden in the general population, the presence of PPM1D mutations in approximately one-fifth of patients with therapy-related hematological neoplasms is suggestive of a substantial role for these alterations in therapy-driven clonal selection (61, 68).

*In vivo* murine models have demonstrated that PPM1D regulates the competitive fitness and self-renewal capacity of HSCs, even in the absence of genotoxic stress, thus supporting the idea that that the biological effects of this gene are not strictly contingent upon cytotoxic therapy (69). Despite these insights, the exact role of PPM1D in the context of CH and malignant transformation remains to be fully characterized. Their detectability at low VAF levels in healthy individuals suggests that undocumented cytotoxic exposures or alternative selective pressures may contribute to the emergence of these mutations.

Although TP53 is one of the most comprehensively studied DDR genes in the context of hematopoiesis, the underlying biological mechanisms by which its mutations confer a selective advantage in HSCs have not yet been fully elucidated (67). While heterozygous TP53 mutations confer a pronounced selective advantage on mutant cells, particularly following cytotoxic therapy, they can also emerge in CH cases in the absence of any overt selective pressure. Research has demonstrated that the p53 protein is implicated in a multitude of cellular processes, including stem cell differentiation, and that the loss of TP53 imparts a dose-dependent competitive advantage in both young and aged HSCs (70, 71). The impact of TP53 mutations in CH cannot therefore be solely attributed to heightened genomic instability, and researchers have postulated that distinct mechanisms affecting stem cell biology also contribute to this process. In particular, biallelic TP53 loss coupled with cytotoxic therapy exposure significantly increases the risk of malignant transformation (72). Furthermore, the biological behaviors of different TP53 mutation classes exhibit distinct variations, which can in turn exhibit a differential effect on the progression from CH to leukemia (73).

The role of TP53 mutations in therapy-related myeloid neoplasms is supported by an extensive body of research. Analyses of t-AML patients reveal that TP53 mutations are frequently present in the bone marrow at very low VAFs years before the initiation of primary cancer treatment and the onset of clinical disease (67). Indeed, a study evaluating seven t-AML patients harboring a clonal TP53 mutation demonstrated that the mutation driving the t-AML clone was present in the bone marrow years before the initiation of anticancer treatment in four of these cases. Those authors also reported that using ultra-sensitive next-generation sequencing (NGS) methodologies, TP53 mutations were detected at VAFs ranging from 0.01% to 0.37% in nine out of 19 clinically healthy elderly individuals. *In vivo* experiments involving chimeric murine models have also confirmed that TP53-mutant cells acquire a distinct survival and proliferative advantage following cytotoxic chemotherapy (67). The expansion of TP53-mutant clones, as evidenced by increased VAF levels following cytotoxic therapy, is strongly correlated with the development of therapy-related acute myeloid leukemia and adverse clinical outcomes (67, 74).

Additionally, mutations detected in spliceosome genes, such as SRSF2 and SF3B1, have been closely associated with previous anticancer treatment. These findings suggest that cytotoxic therapies may exert a selective pressure that favors not only DNA damage response genes, but also genes involved in RNA processing (4).

## 5 Effects of cancer treatments on clonal hematopoiesis

### 5.1 Chemotherapy-related effects

Therapy-related myeloid neoplasms are a fatal consequence of exposure to cytotoxic chemotherapy during cancer treatment, and account for approximately 15% of all myeloid neoplasm cases (75, 76).

Cytotoxic agents widely used in cancer treatment, such as platinum-based drugs, exert a strong selective pressure on HSPCs within the bone marrow. Hematopoietic clones harboring mutations in DNA damage response (DDR) genes, such as TP53, which are present at very low levels (below the limit of detection) prior to chemotherapy, are resistant to the genotoxic stress induced by platinum therapy. While normal cells are eradicated by chemotherapy, these mutant clones gain a selective survival and proliferative advantage, expanding rapidly. This dynamic represents the primary driving force behind the development of t-MNs (77). Following platinum-based chemotherapy in patients with small cell lung cancer, CH mutations in DDR pathway genes, such as TP53, PPM1D, and CHEK2, were detected in 57% of patients in the placebo group in another study. Furthermore, the growth rates of clones harboring DDR mutations during platinum-based chemotherapy were significantly higher compared to a control group that did not receive chemotherapy (77).

In the 5th edition of the World Health Organization (WHO) classification (2022), patients exposed to PARP inhibitors were formally included together with those exposed to chemotherapy and radiotherapy within the definition of “myeloid neoplasms post-cytotoxic therapy” (2). This inclusion underscores the clinical recognition of the leukemogenic potential of PARP inhibitor therapy. In recent years, PARPs have been increasingly applied in the management of solid tumors, particularly ovarian and breast cancers (78). The reported cumulative incidence of t-MN development in women with epithelial ovarian cancer treated with PARP inhibitors was 6.9% in the initial study and 8.7% in the subsequent expanded study (79). Although a lower t-MN incidence of approximately 1% was reported among ovarian cancer patients treated with PARP inhibitors in another study, a heightened relative risk was nonetheless confirmed (80). Cases of t-MN developing following PARP inhibitor exposure harbor a highly adverse and refractory genetic signature. According to Marmouset et al. and Martin et al., complex karyotypes and/or TP53 mutations have been identified in over 60% of patients who developed t-MN following PARP inhibitor therapy (81, 82). Unfortunately, the prognosis for t-MN cases developing after PARP inhibitor therapy is remarkably poor. Studies have reported that the median OS for patients who develop t-MN following exposure to PARP inhibitors is critically short, ranging from only 4.3 to five months (83).

While t-MNs are traditionally associated with hematological malignancies, due to the higher epidemiological prevalence of solid cancers, the numbers of t-MN cases developing following solid tumor treatment are not inconsiderable. In that context, DNA-damaging chemotherapeutic agents, particularly platinum-based regimens, and PARP inhibitors exert a profound selective pressure on HSCs.

The conventional threshold of a variant allele frequency (VAF) ≥2% for defining clinically significant CH may be inadequate in the specific setting of cytotoxic selection. Current evidence indicates that clinically “silent” clones with a VAF well below 2% can expand rapidly and drive t-MN transformation in patients treated with platinum-based chemotherapy (84). This highlights the critical need for ultra-sensitive sequencing methodologies, since even exceedingly small mutant clones possess substantial malignant potential under specific therapeutic pressures.

### 5.2 Clonal hematopoiesis in stem cell transplantation

In patients undergoing high-dose chemotherapy and autologous hematopoietic stem cell transplantation (auto-HSCT), cells harboring CH are harvested during leukapheresis, thus being shielded from the direct cytotoxic effects of myeloablative conditioning regimens such as melphalan. Following reinfusion post-transplantation, these cells can rapidly expand by acquiring a selective proliferative advantage, driven by the depleted hematopoietic niches created by chemotherapy within the bone marrow, alongside the immunosuppressive and pro-inflammatory microenvironment. Nevertheless, the heterogeneous expansion rates of mutant clones among individuals and the fact that overt leukemia develops in only a subset of patients suggest that environmental and extrinsic selective pressures, in addition to stochastic genetic events, play a crucial role in the clonal evolution process (85). Determining the role of the bone marrow microenvironment and the extrinsic environmental factors that govern the emergence and clonal expansion of CH is therefore attracting growing interest (86).

Data from human HSC transplantation studies show that these extrinsic factors can represent critical determinants of clonal expansion (87, 88). Particularly in the context of sibling-donor allogeneic HSC transplantation, the observation that donor-derived CH clones reach greater clonal burdens in the recipient than in the donor suggests that the post-transplant bone marrow microenvironment and the reconstituting hematopoietic system actively support the selection of mutant clones (89). Although these observations are based on a limited number of cases, the allogeneic transplantation setting provides invaluable insights into the biology of CH and the mechanisms driving clonal expansion (89).

In the autologous transplant setting, the clinical significance of CH becomes particularly pronounced in terms of the development of t-MNs. The presence of CH clones harboring mutations in genes involved in DDR pathways, most notably PPM1D and TP53, has been identified as a robust and independent predictor of post-transplantation t-MN development (90, 91). Furthermore, a comprehensive meta-analysis also demonstrated that the presence of persisting or engrafting clonal hematopoiesis following autologous hematopoietic stem cell transplantation (auto-HSCT) is associated with a significantly elevated risk of t-MN development, in addition to shortened OS and progression-free survival (PFS) (92). This heightened risk is especially pronounced in patients harboring CH clones with mutations in genes responsible for DNA damage repair and the maintenance of genomic stability, such as PPM1D, TP53, RAD21, and BRCC3, thus representing a clinically high-risk subgroup for the development of secondary myeloid neoplasms (93).

The clinical implications of CH in patients undergoing auto-HSCT have been more clearly elucidated in large patient cohorts. One large-scale, single-center study involving 1,507 patients who underwent auto-HSCT reported that PPM1D and TP53 mutations detected within the context of CH of post-transplant t-MNs (90). Similarly, a recent retrospective analysis evaluating 1,931 lymphoma and multiple myeloma patients treated with auto-HSCT revealed that the presence of CHIP represents a significant risk factor, not only for t-MN, but also for the development of non-myeloid secondary malignant neoplasms. Notably, the risk of developing non-myeloid secondary malignancies was significantly elevated in patients harboring CHIP clones with a high VAF or TP53 mutations (91).

The persistence of CH following treatment is also of clinical importance, particularly concerning the interpretation of minimal residual disease (MRD) in patients with AML. CH-associated mutations, such as those in DNMT3A, TET2, ASXL1, SRSF2, SF3B1, U2AF1, and JAK2, have frequently been shown to persist in patients with AML cases achieving complete remission following standard induction and consolidation chemotherapy. However, the post-treatment persistence of these mutations is not, in isolation, considered an indicator of an adverse prognosis or residual leukemic disease. In contrast, other studies have reported that these CH clones are largely eradicated following allogeneic hematopoietic stem cell transplantation (allo-HSCT); this in turn suggests that allogeneic transplantation is capable of eliminating, not only the leukemic clones, but also the underlying CH clones (94, 95).

A recent meta-analysis compared the outcomes of allo-HSCT and auto-HSCT in the setting of CH engraftment. The analysis demonstrated that the presence of donor-derived CH following allo-HSCT was associated with a decrease in disease relapse. However, it had no significant impact on OS, PFS, or non-relapse mortality. In contrast, the presence of CH in patients undergoing auto-HSCT was correlated with shorter OS and PFS, together with a markedly elevated risk of t-MN development (96). In corroboration of these findings, a second meta-analysis reported that CH-positive patients undergoing auto-HSCT exhibited inferior peripheral blood stem cell mobilization and prolonged platelet engraftment times. These results suggest that CH may adversely affect, not only long-term oncological outcomes, but also the early hematopoietic recovery processes that dictate transplant success (93).

In their evaluation of clonal behavior following allogeneic transplantation, Boettcher et al. investigated CH dynamics in 42 long-term surviving allo-HSCT donor-recipient pairs. In four out of five cases (80%) in which donor-derived CH was detected, the clonal burden in the recipients, as measured by VAF, was shown to have expanded compared to the donors. Nevertheless, the modest extent of the observed clonal expansion suggests that the recipient’s hematopoiesis was not dominated by a single mutant stem cell clone. Furthermore, the development of myelodysplastic syndrome (MDS) during follow-up in both a CH-harboring donor and the corresponding recipient was highlighted as a compelling observation, strongly suggesting that donor-derived CH can precipitate clinically significant consequences under specific conditions (89).

### 5.3 CAR-T therapy

Current evidence indicates that, rather than inducing *de novo* CH mutations, CAR-T cell therapy establishes a microenvironment conducive to the selective proliferation and clonal expansion of pre-existing mutant clones. The profound systemic inflammation, immune dysregulation, and hematopoietic stress that develop during treatment are regarded as the fundamental drivers of this process. Consistent with preclinical studies, the microenvironment orchestrated by inflammatory cytokines suppresses normal hematopoiesis, while simultaneously conferring a marked survival and proliferative advantage, particularly on CH clones harboring TP53 mutations, thus promoting clonal selection within the bone marrow.

The most striking aspect of these profound alterations in the immune microenvironment is that CAR-T therapy does not uniformly expand all CH clones, but rather specifically fosters the selective expansion of TP53-mutant clones. Researchers have postulated that the hematological toxicity, intense immune activation, and inflammatory stress secondary to CAR-T therapy exert a robust selective pressure favoring TP53-mutant clones within the bone marrow. This biological process facilitates the dominance of high-risk clones and is regarded as a pivotal mechanism that may contribute to the subsequent development of secondary myeloid neoplasms, such as MDS or AML, in a subset of patients (97).

### 5.4 Radiotherapy

Radiotherapy is recognized as a prominent therapy-induced selective pressure capable of influencing the development of CH. Cancer patients undergoing radiotherapy have been reported to exhibit an approximately two-fold increased risk of developing CH, specifically those harboring DNMT3A mutations, compared to the general population. A large-scale study evaluating 772 patients who had received radiotherapy at least six months prior to blood sampling reported that mutations in at least one CH-associated gene were detected in 5.1% of the cohort. Subgroup analyses stratified according to primary tumor localization revealed a significantly higher propensity for CH development in patients receiving radiotherapy for endometrial and vulvar cancers, as well as for hematological malignancies such as leukemia and multiple myeloma. Conversely, no significant correlation was established between the cumulative radiation dose, calculated as the equivalent dose in 2 Gy fractions (EQD2), and the incidence of CH (98).

In addition to external beam radiotherapy, the recent introduction of radioligand therapies, which are becoming increasingly used in the management of prostate and neuroendocrine cancers, has introduced a new class of therapeutic selective pressure.

The current literature shows that radioligand therapies are significantly associated with an increased risk of newly emerging therapy-related CH (and subsequent t-MN development. The profound impact of these increasingly widely used therapies necessitates close monitoring for clonal evolution in these growing patient populations.

A study comparing the prevalence of CH following novel radioligand therapy with the alternative standard-of-care cabazitaxel chemotherapy in metastatic castration-resistant prostate cancer reported a higher frequency of CH with radioligand therapy compared to cabazitaxel. PPM1D mutations were most frequently observed among DDR genes, accounting for 42% of cases. These findings strongly support the integration of longitudinal monitoring for long-term hematological sequelae, particularly since radioligand therapy is increasingly being implemented earlier in the prostate cancer treatment paradigm (99).

In another study suggesting that the hematological toxic effects of peptide receptor radionuclide therapy (PRRT) can be persistent, patients with neuroendocrine tumors developed persistent cytopenias following PRRT exposure. The expansion of mutant TP53, CHEK2, and PPM1D clones was also observed concurrently with the onset of these cytopenias (100).

## 6 Discussion

Novel models developed in recent years to predict the risk of myeloid malignancy in individuals with CH are intended to better elucidate the clinical significance of these silent clones. The CHRS seeks to identify high-risk individuals by incorporating age, the presence of cytopenias, mutational patterns, and hematological parameters. Similarly, the MN-predict model estimates the long-term risk of myeloid neoplasia by integrating genotypic, phenotypic, and biochemical data. In the future, these prognostic models may facilitate the standardized monitoring of patients with CHIP and clonal cytopenia of undetermined significance, thus contributing to the early identification of high-risk individuals.

Research has demonstrated that in a significant proportion of adult patients who develop t-MNs following oncological treatment, CH mutations present in the circulation prior to primary cancer therapy undergo selective clonal expansion over time, transforming into dominant malignant clones (4). The genotoxic stress induced by cytotoxic therapies exerts a strong selection pressure on these pre-existing resistant clones, thereby accelerating the process of malignant transformation.

The molecular basis of this selection pressure is explained by the fact that different anticancer therapies confer a specific proliferative advantage on CH clones harboring particular mutations. By evaluating CH dynamics in a large cancer cohort undergoing chemotherapy and radiotherapy, Bolton et al. demonstrated that antineoplastic therapies are not only mutagenic agents, but can also act as critical driver factors that select for pre-existing CH clones. The authors also reported that SRSF2 and SF3B1 mutations were strongly associated with prior anticancer therapies, while ASXL1 mutations were highly correlated with smoking (4).

Among the various anticancer treatment modalities, radiotherapy, radionuclide therapy, and cytotoxic chemotherapy, in particular, exhibit the strongest association with CH-harboring myeloid driver gene mutations (101). These findings are further supported by sequential genetic analyses involving 525 patients with solid tumors who received cytotoxic chemotherapy and/or external radiotherapy (61%) or targeted therapy/immunotherapy (31%), the presence of CH being detected in 74% of these cases (4).

The critical role of TP53 mutations in the development of t-MNs is emphasized by the detection of these mutations at low VAF levels years before disease diagnosis. The marked expansion of TP53-mutant clones following cytotoxic therapy suggests that the treatment primarily selects for pre-existing resistant clones, rather than inducing *de novo* mutations. Similarly, PPM1D mutations confer a robust clonal advantage, particularly following chemotherapy exposure, emerging as a crucial biological determinant in the pathogenesis of t-MNs. The observation that PPM1D-mutant clones may initially exist at very small clone sizes implies that these can elude traditional surveillance methods, emphasizing that their “silent” nature does not equate to biological insignificance.

In contrast, mutations in epigenetic regulatory genes, such as TET2 and DNMT3A, exhibit a distinct pattern of clonal behavior. Loss of function in TET2 enhances the self-renewal capacity of HSCs and drives the selective expansion of mutant clones through complex interactions with the inflammatory microenvironment. In particular, proinflammatory cytokines such as IL-1β, IL-6, and TNF-α have been shown to establish a positive feedback loop that bolsters the survival of TET2-mutant cells. This phenomenon emphasizes that CH is not exclusively a genetic event, but in fact a dynamic evolutionary process sculpted by the inflammatory microenvironment.

DNMT3A mutations constitute one of the most frequently observed genetic alterations within CHIP, driving a reduction in the differentiation capacity of HSCs while at the same time enhancing their self-renewal potential. However, a solitary DNMT3A mutation is generally not considered sufficient to precipitate malignant transformation. Instead, the acquisition of additional genetic events or the imposition of environmental stressors are required. The clinical evaluation of DNMT3A-mutant clones must therefore extend beyond the mere presence of the mutation, necessitating a comprehensive assessment of the specific mutation type, the VAF level, and any concurrent genetic alterations.

Longitudinal studies have demonstrated that different chemotherapeutic agents exert selective pressure on clones harboring specific gene mutations. Specifically, platinum compounds, radionuclide therapies, and topoisomerase I/II inhibitors significantly promote the expansion of clones harboring mutations in genes involved in DDR pathways, such as TP53 and PPM1D (67, 102, 103). Similarly, lenalidomide, an agent widely used in the treatment of multiple myeloma, induces the ubiquitination and degradation of the CK1α protein, which normally triggers apoptosis by enabling p53 activation. However, cells harboring TP53 mutations bypass this apoptotic mechanism, thus acquiring a significant survival and proliferation advantage under lenalidomide therapy (104, 105). In recent years, next-generation targeted therapies, such as peptide receptor radionuclide therapy and poly (ADP-ribose) polymerase inhibitors, have also been reported to similarly modulate the cellular stress response, promoting the selective expansion of clones specifically carrying TP53 and PPM1D mutations, which may ultimately contribute to the development of t-MNs (100, 106).

Cancer-associated CH is not only closely associated with therapy-related myeloid malignancies, but also with the overall development of secondary hematological malignancies. Even silent variants, which do not alter the protein sequence, have been shown to be of predictive value for this progression. Furthermore, the presence of CH, detected in approximately 29.7% of patients with solid tumors, has been reported to approximately double the risk of developing a therapy-related hematological malignancy (107).

In order for leukemic transformation to occur, however, the acquisition of additional genetic events beyond the TP53 mutation, or the presence of a strong selection pressure supporting the expansion of TP53-mutant clones, is frequently required. This phenomenon is particularly evident in cancer patients undergoing chemotherapy and/or radiotherapy. The resulting genomic instability at the final stage manifests with characteristic cytogenetic alterations. Exposure to alkylating agents has been shown to be strongly associated with 5q and 7q deletions, observed in approximately 76% of therapy-related myeloid neoplasm cases presenting with an abnormal karyotype (108). In contrast, in therapy-related acute myeloid leukemias (t-AML) associated with topoisomerase inhibitors, characteristic translocations involving the 11q23 and 21q22 chromosomal regions have been detected in approximately 5–20% of cases (109, 110).

The inflammatory environment associated with CH not only acts as a precursor to hematological malignancies, but can also significantly impact solid tumor outcomes. Recent research into tumor-infiltrating CH has revealed that mutant myeloid cells can migrate into the solid tumor microenvironment, modulate local immune responses, and potentially alter the cancer trajectory and therapeutic efficacy.

Following the recognition of these mutant clones, clinical interest in the therapeutic targeting of CH, specifically for the prevention of t-MN, is growing rapidly. As highlighted in recent pivotal presentations, novel targeted interventions aimed at suppressing specific mutant clones or mitigating their inflammatory drivers are currently being investigated. The transition from passive observation to proactive intervention holds tremendous promise in terms of altering the natural course of therapy-related myeloid neoplasms.

The post-HSCT setting represents one of the most compelling clinical models illustrating the biological significance of silent clones. In patients undergoing autologous HSCT, pre-existing CHIP clones can undergo marked expansion, driven by the selective pressure exerted by conditioning chemotherapy and the profoundly altered post-transplant bone marrow microenvironment, thereby predisposing the patient to the development of t-MNs. Notably, mutations in TP53 and PPM1D have been identified as robust predictors of the risk of post-transplantation myeloid neoplasia. These findings suggest that pre-transplant molecular screening may be of significant value, as both a prognostic tool and as a critical cornerstone for the development of preventative clinical strategies.

In the future, the mere detection of existing mutations will be insufficient for an accurate t-MN risk assessment. A comprehensive evaluation must concurrently integrate clone size, clonal growth kinetics, the functional impact of the specific mutations, interactions with the inflammatory microenvironment, and post-therapeutic clonal dynamics. Furthermore, the application of ultrasensitive next-generation sequencing methodologies and single-cell analysis technologies will facilitate the early identification of clinically silent clones that harbor a high malignant potential.

In order to translate these preventive concepts into clinical practice, several active clinical trials are currently investigating targeted interventions for individuals with high-risk CH or CCUS. For instance, ongoing studies (10) are evaluating the safety and efficacy of novel therapeutic agents designed to halt clonal expansion and prevent cytopenia progression. Future research should focus on prospective, randomized controlled trials to determine whether early molecular screening and pharmacological intervention, such as targeted mutation inhibitors or immune modulators, in patients harboring high-risk t-CH clones can effectively delay or prevent the onset of therapy-related myeloid neoplasms. Crucially, these future strategies must be carefully balanced to ensure that targeted CH interventions do not compromise the curative efficacy of the primary cancer treatments.

A recently published study presented a groundbreaking pharmacological strategy and future perspectives for targeting CH clones, particularly DDR pathway mutations such as TP53, to prevent t-MN. That research showed that transient CDK4/6 inhibition (e.g., using trilaciclib or palbociclib) administered prior to platinum therapy effectively prevents the adverse effects induced by platinum-based chemotherapy. The authors emphasized that the success rate of therapies administered after the onset of t-MN are exceedingly low, and that the disease is almost universally fatal. They therefore advocated for a paradigm of upfront prevention rather than relying on treatment. Suppressing CH clonal expansion during cytotoxic chemotherapy, representing the earliest stage of t-MN development, has been proposed as the highest-yield strategy for eradicating this fatal disease. Mechanistically, while CDK4/6 inhibition induces a protective quiescent state (G0/G1 phase arrest) in normal HSCs, it triggers apoptosis (programmed cell death) in Trp53-mutant stem cells, thereby abrogating their platinum-induced stemness advantage. Normal stem cells are thus preserved, while TP53-mutant clones undergo apoptosis. Twenty-four active clinical trials investigating the combination of trilaciclib with various cytotoxic chemotherapy regimens are currently reported to be underway. That article anticipates that evaluating the results of these trials will yield critical insights into the safety of CDK4/6 inhibitors and will guide the design of large-scale clinical trials dedicated to the prevention of t-MN (77).

## 7 Conclusion

Considered from a broader perspective, CH emerges as a significant risk factor for the development of t-MNs following cytotoxic chemotherapy, radiotherapy, and autologous hematopoietic stem cell transplantation. Indeed, research has shown that preleukemic CH clones present prior to cancer therapy are frequently detected in patients who subsequently develop t-MN. Current data reveal that cytotoxic therapies exert a strong selection pressure, particularly on HSC clones harboring mutations in genes associated with the DNA damage response, such as PPM1D and TP53, thereby promoting their expansion. The evaluation of CH mutations, especially high-risk TP53 mutations that undergo positive selection during treatment, in cancer patients prior to and during therapy follow-up may contribute to the early identification of patients at a high risk of developing t-MN.

An improved understanding of the biological mechanisms involved in CH may assist the development of more individualized therapeutic approaches in patients at a high risk of treatment-related hematological toxicity and secondary malignancies. In this context, avoiding agents with high leukemogenic potential, considering dose modifications, or selecting alternative therapeutic options may represent an appropriate strategy in patients harboring high-risk CH clones. However, such decisions must be made on a case-by-case basis and through a multidisciplinary approach, carefully weighing the survival benefit derived from the treatment of the primary malignancy against the risk of developing a therapy-related myeloid neoplasm. For example, individualized treatment strategies can be planned by considering the potential effects of lenalidomide therapy, which has been reported to support the selective expansion of TP53-mutant clones in multiple myeloma, in patients carrying high-risk CH.

Current evidence indicates that CHIP is a significant prognostic biomarker not only for hematological malignancies, but also for numerous clinical outcomes. Nevertheless, discrepancies in the definition of CHIP, the sequencing methods used for its detection, VAF thresholds, and reporting criteria complicate the direct translation of these findings into routine clinical practice. Furthermore, the effects on CH of environmental exposures capable of mimicking the cellular stress and selection pressure induced by chemotherapy in healthy individuals have not yet been fully elucidated.

In conclusion, t-MNs should no longer be regarded merely as secondary malignancies arising post-treatment. Instead, they must be recognized as the culmination of protracted, silently progressing clonal evolutionary processes that frequently originate years previously. The concept of the “power of silent clones” provides a novel biological perspective in t-MN risk stratification, establishing a foundational framework for the future development of personalized surveillance, early intervention, and targeted preventative strategies. Elucidating the profound molecular underpinnings of CH will not only enable the precise identification of high-risk individuals, but will also pave the way for the development of innovative therapeutic approaches aimed at intercepting myeloid malignancies prior to their clinical onset.

## References

[1] McClatchy J Strogantsev R Wolfe E Lin HY Mohammadhosseini M Davis BA . Clonal hematopoiesis related TET2 loss-of-function impedes IL1β-mediated epigenetic reprogramming in hematopoietic stem and progenitor cells. Nat Commun. (2023) 14(1):8102. doi: 10.1038/s41467-023-43697 38062031 PMC10703894

[2] Khoury JD Solary E Abla O Akkari Y Alaggio R Apperley JF . The 5th edition of the World Health Organization classification of haematolymphoid tumours: myeloid and histiocytic/dendritic neoplasms. Leukemia. (2022) 36:1703–19. doi: 10.1038/s41375-022-01613-1 35732831 PMC9252913

[3] Arber DA Orazi A Hasserjian RP Borowitz MJ Calvo KR Kvasnicka HM . International Consensus Classification of myeloid neoplasms and acute leukemias: integrating morphologic, clinical, and genomic data. Blood. (2022) 140:1200–28. doi: 10.1182/blood.2022015850 35767897 PMC9479031

[4] Bolton KL Ptashkin RN Gao T Braunstein L Devlin SM Kelly D . Cancer therapy shapes the fitness landscape of clonal hematopoiesis. Nat Genet. (2020) 52:1219–26. doi: 10.1038/s41588-020-00710-0 33106634 PMC7891089

[5] Takahashi K Wang F Kantarjian H Doss D Khanna K Thompson E . Preleukaemic clonal haemopoiesis and risk of therapy-related myeloid neoplasms: a case-control study. Lancet Oncol. (2017) 18:100–11. doi: 10.1016/s1470-2045(16)30626-x 27923552 PMC5405697

[6] Desai P Mencia-Trinchant N Savenkov O Simon MS Cheang G Lee S . Somatic mutations precede acute myeloid leukemia years before diagnosis. Nat Med. (2018) 24:1015–23. doi: 10.1038/s41591-018-0081-z 29988143 PMC6849383

[7] Weeks LD Niroula A Neuberg D Wong W Lindsley K Luskin MR . Prediction of risk for myeloid malignancy in clonal hematopoiesis. NEJM Evid. (2023) 2(5):EVIDoa2200310. doi: 10.1056/evidoa2200310 37483562 PMC10361696

[8] Gu M Kovilakam SC Dunn WG Marando L Barcena C Mohorianu I . Multiparameter prediction of myeloid neoplasia risk. Nat Genet. (2023) 55:1523–30. doi: 10.1038/s41588-023-01472-1 37620601 PMC10484784

[9] Xie Z Komrokji R Al Ali N Regelson A Geyer S Patel A . Risk prediction for clonal cytopenia: multicenter real-world evidence. Blood. (2024) 144(19):2033–44. doi: 10.1182/blood.2024024756 38996210 PMC11561536

[10] Weeks LD . A multi-site break through cancer pilot study testing the feasibility and safety of therapeutic intervention for patients with high-risk clonal cytopenia of undetermined significance (CCUS) Clinicaltrials.Gov Identifier: NCT06802146. Bethesda, MD: National Library of Medicine (US). (2026).

[11] Elena C Gallì A Bono E Todisco G Malcovati L . Clonal hematopoiesis and myeloid malignancies: clonal dynamics and clinical implications. Curr Opin Hematol. (2021) 28:347–55. doi: 10.1097/moh.0000000000000675 34342292

[12] Costa A Pilo F Pettinau M Piras E Targhetta C Rojas R . Impact of primary cancer history and molecular landscape in therapy-related myeloid neoplasms. Front Oncol. (2025) 15:1563990. doi: 10.3389/fonc.2025.1563990 40342822 PMC12058663

[13] Hajishengallis G Chavakis T . Inflammaging and clonal hematopoiesis interplay and their impact on human disease. Nat Rev Mol Cell Biol. (2026) 27:377–93. doi: 10.1038/s41580-025-00936-y 41491423 PMC12795708

[14] Caiado F Kovtonyuk LV Gonullu NG Fullin J Boettcher S Manz MG . Aging drives Tet2+/- clonal hematopoiesis via IL-1 signaling. Blood. (2023) 141:886–903. doi: 10.1182/blood.2022016835 36379023 PMC10651783

[15] Jacobsen SEW Nerlov C . Haematopoiesis in the era of advanced single-cell technologies. Nat Cell Biol. (2019) 21:2–8. doi: 10.1038/s41556-018-0227-8 30602765

[16] Chavez JS Rabe JL Loeffler D Higa KC Hernandez G Mills TS . PU.1 enforces quiescence and limits hematopoietic stem cell expansion during inflammatory stress. J Exp Med. (2021) 218(6):e20201169. doi: 10.1084/jem.20201169 33857288 PMC8056754

[17] Pietras EM Mirantes-Barbeito C Fong S Loeffler D Kovtonyuk LV Zhang S . Chronic interleukin-1 exposure drives haematopoietic stem cells towards precocious myeloid differentiation at the expense of self-renewal. Nat Cell Biol. (2016) 18:607–18. doi: 10.1038/ncb3346 27111842 PMC4884136

[18] Fuster JJ MacLauchlan S Zuriaga MA Polackal MN Ostriker AC Chakraborty R . Clonal hematopoiesis associated with TET2 deficiency accelerates atherosclerosis development in mice. Science. (2017) 355:842–7. doi: 10.1126/science.aag1381 28104796 PMC5542057

[19] Kaasinen E Kuismin O Rajamäki K Ristolainen H Aavikko M Kondelin J . Impact of constitutional TET2 haploinsufficiency on molecular and clinical phenotype in humans. Nat Commun. (2019) 10:1252. doi: 10.1038/s41467-019-09198-7 30890702 PMC6424975

[20] Abegunde SO Buckstein R Wells RA Rauh MJ . An inflammatory environment containing TNFα favors Tet2-mutant clonal hematopoiesis. Exp Hematol. (2018) 59:60–5. doi: 10.1016/j.exphem.2017.11.002 29195897

[21] Busque L Patel JP Figueroa ME Vasanthakumar A Provost S Hamilou Z . Recurrent somatic TET2 mutations in normal elderly individuals with clonal hematopoiesis. Nat Genet. (2012) 44:1179–81. doi: 10.1038/ng.2413 23001125 PMC3483435

[22] SanMiguel JM Eudy E Loberg MA Young KA Mistry JJ Mujica KD . Distinct tumor necrosis factor alpha receptors dictate stem cell fitness versus lineage output in dnmt3a-mutant clonal hematopoiesis. Cancer Discov. (2022) 12:2763–73. doi: 10.1158/2159-8290.CD-22-0086 36169447 PMC9716249

[23] Cai Z Kotzin JJ Ramdas B Chen S Nelanuthala S Palam LR . Inhibition of inflammatory signaling in Tet2 mutant preleukemic cells mitigates stress-induced abnormalities and clonal hematopoiesis. Cell Stem Cell. (2018) 23:833–49.e5. doi: 10.1016/j.stem.2018.10.013 30526882 PMC6317370

[24] Cull AH Snetsinger B Buckstein R Wells RA Rauh MJ . Tet2 restrains inflammatory gene expression in macrophages. Exp Hematol. (2017) 55:56–70. doi: 10.1016/j.exphem.2017.08.001 28826859

[25] Zhang Q Zhao K Shen Q Han Y Gu Y Li X . Tet2 is required to resolve inflammation by recruiting Hdac2 to specifically repress IL-6. Nature. (2015) 525:389–93. doi: 10.1038/nature15252 26287468 PMC4697747

[26] Biagi E Candela M Franceschi C Brigidi P . The aging gut microbiota: new perspectives. Ageing Res Rev. (2011) 10:428–9. doi: 10.1016/j.arr.2011.03.004 21402177

[27] Meisel M Hinterleitner R Pacis A Chen L Earley ZM Mayassi T . Microbial signals drive pre-leukaemic myeloproliferation in a Tet2-deficient host. Nature. (2018) 557:580–4. doi: 10.1038/s41586-018-0125-z 29769727 PMC6238954

[28] Bick AG Pirruccello JP Griffin GK Gupta N Gabriel S Saleheen D . Genetic interleukin 6 signaling deficiency attenuates cardiovascular risk in clonal hematopoiesis. Circulation. (2020) 141:124–31. doi: 10.1161/circulationaha.119.044362 31707836 PMC7008855

[29] Zhang CRC Nix D Gregory M Ciorba MA Ostrander EL Newberry RD . Inflammatory cytokines promote clonal hematopoiesis with specific mutations in ulcerative colitis patients. Exp Hematol. (2019) 80:36–41. doi: 10.1016/j.exphem.2019.11.008 31812712 PMC7031927

[30] Tahiliani M Koh KP Shen Y Pastor WA Bandukwala H Brudno Y . Conversion of 5-methylcytosine to 5- hydroxymethylcytosine in mammalian DNA by MLL partner TET1. Science. (2009) 324:930–5. doi: 10.1126/science.1170116 19372391 PMC2715015

[31] Ito S D’Alessio AC Taranova OV Hong K Sowers LC Zhang Y . Role of Tet proteins in 5mC to 5hmC conversion, ES-cell self-renewal and inner cell mass specification. Nature. (2010) 466:1129–33. doi: 10.1038/nature09303 20639862 PMC3491567

[32] Rasmussen KD Jia G Johansen JV Pedersen MT Rapin N Bagger FO . Loss of TET2 in hematopoietic cells leads to DNA hypermethylation of active enhancers and induction of leukemogenesis. Genes Dev. (2015) 29:910–22. doi: 10.1101/gad.260174.115 25886910 PMC4421980

[33] Gu T Lin X Cullen SM Luo M Jeong M Estecio M . DNMT3A and TET1 cooperate to regulate promoter epigenetic landscapes in mouse embryonic stem cells. Genome Biol. (2018) 19:88. doi: 10.1186/s13059-018-1464-7 30001199 PMC6042404

[34] Ostrander EL Kramer AC Mallaney C Celik H Koh WK Fairchild J . Divergent effects of Dnmt3a and Tet2 mutations on hematopoietic progenitor cell fitness. Stem Cell Rep. (2020) 14:551–60. doi: 10.1016/j.stemcr.2020.02.011 32220332 PMC7160307

[35] Odejide O Weigert O Lane AA Toscano D Lunning MA Kopp N . A targeted mutational landscape of angioimmunoblastic T-cell lymphoma. Blood. (2014) 123:1293–6. doi: 10.1182/blood-2013-10-531509 24345752 PMC4260974

[36] Abdel-Wahab O Mullally A Hedvat C Garcia-Manero G Patel J Wadleigh M . Genetic characterization of TET1, TET2, and TET3 alterations in myeloid malignancies. Blood. (2009) 114:144–7. doi: 10.1182/blood-2009-03-210039 19420352 PMC2710942

[37] Delhommeau F Dupont S Della Valle V James C Trannoy S Massé A . Mutation in TET2 in myeloid cancers. N Engl J Med. (2009) 360:2289–301. doi: 10.1056/nejmoa0810069 19474426

[38] Li Z Cai X Cai CL Wang J Zhang W Petersen BE . Deletion of Tet2 in mice leads to dysregulated hematopoietic stem cells and subsequent development of myeloid malignancies. Blood. (2011) 118:4509–18. doi: 10.1182/blood-2010-12-325241 21803851 PMC3952630

[39] Buscarlet M Provost S Zada YF Barhdadi A Bourgoin V Lépine G . DNMT3A and TET2 dominate clonal hematopoiesis and demonstrate benign phenotypes and different genetic predispositions. Blood. (2017) 130:753–62. doi: 10.1182/blood-2017-04-777029 28655780

[40] Moran-Crusio K Reavie L Shih A Abdel-Wahab O Ndiaye-Lobry D Lobry C . Tet2 loss leads to increased hematopoietic stemcell self-renewal and myeloid transformation. Cancer Cell. (2011) 20:11–24. doi: 10.1016/j.ccr.2011.06.001 21723200 PMC3194039

[41] Zeng H He H Guo L Li J Lee M Han W . Antibiotic treatment ameliorates Ten-eleven translocation 2 (TET2) loss-of-function associated hematological malignancies. Cancer Lett. (2019) 467:1–8. doi: 10.1016/j.canlet.2019.09.013 31563562 PMC6945766

[42] Heyde A Rohde D McAlpine CS Zhang S Hoyer FF Gerold JM . Increased stem cell proliferation in atherosclerosis accelerates clonal hematopoiesis. Cell. (2021) 184:1348–61. doi: 10.1016/j.cell.2021.01.049 33636128 PMC8109274

[43] Chen T Ueda Y Dodge JE Wang Z Li E . Establishment and maintenance of genomic methylation patterns in mouse embryonic stem cells by Dnmt3a and Dnmt3b. Mol Cell Biol. (2003) 23:5594–603. doi: 10.1128/mcb.23.16.5594-5605.2003 12897133 PMC166327

[44] Yang L Rau R Goodell MA . DNMT3A in haematological malignancies. Nat Rev Cancer. (2015) 15:152–65. doi: 10.1038/nrc3895 25693834 PMC5814392

[45] Challen GA Sun D Jeong M Luo M Jelinek J Berg JS . Dnmt3a is essential for hematopoietic stem cell differentiation. Nat Genet. (2011) 44:23–31. doi: 10.1182/blood.v118.21.386.386 PMC3637952 22138693

[46] Jeong M Park HJ Celik H Ostrander EL Reyes JM Guzman A . Loss of Dnmt3a immortalizes hematopoietic stem cells *in vivo*. Cell Rep. (2018) 23:1–10. doi: 10.1016/j.celrep.2018.03.025 29617651 PMC5908249

[47] Challen GA Sun D Mayle A Jeong M Luo M Rodriguez B . Dnmt3a and Dnmt3b have overlapping and distinct functions in hematopoietic stem cells. Cell Stem Cell. (2014) 15:350–64. doi: 10.1016/j.stem.2014.06.018 25130491 PMC4163922

[48] Spencer DH Russler-Germain DA Ketkar S Helton NM Lamprecht TL Fulton RS . CpG island hypermethylation mediated by DNMT3A is a consequence of AML progression. Cell. (2017) 168:801–13. doi: 10.1016/j.cell.2017.01.021 28215704 PMC5328582

[49] Xu J Wang YY Dai YJ Zhang W Zhang WN Xiong SM . DNMT3A Arg882 mutation drives chronic myelomonocytic leukemia through disturbing gene expression/ DNA methylation in hematopoietic cells. Proc Natl Acad Sci USA. (2014) 111:2620–5. doi: 10.1073/pnas.1400150111 24497509 PMC3932885

[50] Jaiswal S Fontanillas P Flannick J Manning A Grauman PV Mar BG . Age-related clonal hematopoiesis associated with adverse outcomes. N Engl J Med. (2014) 371:2488–98. doi: 10.1056/NEJMoa1408617 25426837 PMC4306669

[51] Brunetti L Gundry MC Goodell MA . New insights into the biology of acute myeloid leukemia with mutated NPM1. Int J Hematol. (2019) 110:150–60. doi: 10.1007/s12185-018-02578-7 30632059

[52] Kim SJ Zhao H Hardikar S Singh AK Goodell MA Chen T . A DNMT3A mutation common in AML exhibits dominant-negative effects in murine ES cells. Blood. (2013) 122:4086–9. doi: 10.1182/blood-2013-02-483487 24167195 PMC3952368

[53] Russler-Germain DA Spencer DH Young MA Lamprecht TL Miller CA Fulton R . The R882H DNMT3A mutation associated with AML dominantly inhibits wild-type DNMT3A by blocking its ability to form active tetramers. Cancer Cell. (2014) 25:442–54. doi: 10.1016/j.ccr.2014.02.010 24656771 PMC4018976

[54] Challen GA Goodell MA . Clonal hematopoiesis: mechanisms driving dominance of stem cell clones. Blood. (2020) 136:1590–8. doi: 10.1182/blood.2020006510 32746453 PMC7530644

[55] Gibson CJ Kim HT Zhao L Murdock HM Hambley B Ogata A . Donor clonal hematopoiesis and recipient outcomes after transplantation. J Clin Oncol. (2022) 40:189–201. doi: 10.1200/jco.21.02286 34793200 PMC8718176

[56] Van den Akker EB Makrodimitris S Hulsman M Brugman MH Nikolic T Bradley T . Dynamic clonal hematopoiesis and functional T-cell immunity in a supercentenarian. Leukemia. (2021) 35:2125–9. doi: 10.1038/s41375-020-01086-0 PMC8257492 33184493

[57] Cordua S Kjaer L Skov V Pallisgaard N Hasselbalch HC Ellervik C . Prevalence and phenotypes of JAK2 V617F and calreticulin mutations in a Danish general population. Blood. (2019) 134:469–79. doi: 10.1182/blood.2019001113 31217187

[58] Mullally A Poveromo L Schneider RK Al- Shahrour F Lane SW Ebert BL . Distinct roles for long-term hematopoietic stem cells and erythroid precursor cells in a murine model of Jak2V617F-mediated polycythemia vera. Blood. (2012) 120:166–72. doi: 10.1182/blood-2012-01-402396 22627765 PMC3390954

[59] Hasan S Lacout C Marty C Cuingnet M Solary E Vainchenker W . JAK2V617F expression in mice amplifies early hematopoietic cells and gives them a competitive advantage that is hampered by IFNa. Blood. (2013) 122:1464–77. doi: 10.1182/blood-2013-04-498956 23863895

[60] Kent DG Li J Tanna H Fink J Kirschner K Pask DC . Self-renewal of single mouse hematopoietic stem cells is reduced by JAK2V617F without compromising progenitor cell expansion. PloS Biol. (2013) 11:e1001576. doi: 10.1371/journal.pbio.1001576 23750118 PMC3672217

[61] Hsu JI Dayaram T Tovy A De Braekeleer E Jeong M Wang F . PPM1D mutations drive clonal hematopoiesis in response to cytotoxic chemotherapy. Cell Stem Cell. (2018) 23:700–713.e706. doi: 10.1016/j.stem.2018.10.004 30388424 PMC6224657

[62] Wong TN Miller CA Jotte MRM Bagegni N Baty JD Schmidt AP . Cellular stressors contribute to the expansion of hematopoietic clones of varying leukemic potential. Nat Commun. (2018) 9:455. doi: 10.1038/s41467-018-02858-0 29386642 PMC5792556

[63] Wong TN Miller CA Klco JM Petti A Demeter R Helton NM . Rapid expansion of preexisting nonleukemic hematopoietic clones frequently follows induction therapy for de novo AML. Blood. (2016) 127:893–7. doi: 10.1182/blood-2015-10-677021 26631115 PMC4760092

[64] Bowman RL Busque L Levine RL . Clonal hematopoiesis and evolution to hematopoietic malignancies. Cell Stem Cell. (2018) 22:157–70. doi: 10.1016/j.stem.2018.01.011 29395053 PMC5804896

[65] Badar T Marando L Latorre E Lasho T Olivia FD Lin C . Spectrum, prevalence, and clinical correlates of PPM1D mutations in patients with clonal hematopoiesis and clonal cytopenias. Blood Adv. (2026) 10(5):1746–55. doi: 10.1182/bloodadvances.2025018031 41499760 PMC12969418

[66] Kahn JD Miller PG Silver AJ Sellar RS Bhatt S Gibson C . PPM1D-truncating mutations confer resistance to chemotherapy and sensitivity to PPM1D inhibition in hematopoietic cells. Blood. (2018) 132:1095–105. doi: 10.1182/blood-2018-05-850339 29954749 PMC6137556

[67] Wong TN Ramsingh G Young AL Miller CA Touma W Welch JS . Role of TP53 mutations in the origin and evolution of therapy-related acute myeloid leukaemia. Nature. (2015) 518:552–5. doi: 10.1038/nature13968 PMC4403236 25487151

[68] Bonner EA Song A Arriaga-Gomez E Gadek K Galvis LB Linton J . MDS-associated SF3B1 mutations promote aberrant hematopoietic cell fate choice by disrupting mediator kinase module component CDK8. Blood. (2024) 144:344–5. doi: 10.1182/blood-2024-204075

[69] Miller PG Sperling AS Meyerhofer C McConkey ME Ellegast JM Silva CD . PPM1D modulates. Hematopoietic cell fitness and response to DNA damage and is a therapeutic target in myeloid malignancy. Blood. (2023) 142:2079–88. doi: 10.1182/blood.2023020331 37595362 PMC10733824

[70] Dumble M Moore L Chambers SM Geiger H Van Zant G Goodell MA . The impact of altered p53 dosage on hematopoietic stem cell dynamics during aging. Blood. (2007) 109:1736–42. doi: 10.1182/blood-2006-03-010413 PMC1794064 17032926

[71] Bondar T Medzhitov R . p53-mediated hematopoietic stem and progenitor cell competition. Cell Stem Cell. (2010) 6:309–22. doi: 10.1016/j.stem.2010.03.002 20362536 PMC2872065

[72] Haase D Stevenson KE Neuberg D Maciejewski JP Nazha A Sekeres MA . TP53 mutation status divides myelodysplastic syndromes with complex karyotypes into distinct prognostic subgroups. Leukemia. (2019) 33:1747–58. doi: 10.1038/s41375-018-0351-2 30635634 PMC6609480

[73] Boettcher S Miller PG Sharma R McConkey M Leventhal M Krivtsov AV . A dominant-negative effect drives selection of TP53 missense mutations in myeloid malignancies. Science. (2019) 365:599–604. doi: 10.1126/science.aax3649 31395785 PMC7327437

[74] Chen S Wang Q Yu H Capitano ML Vemula S Nabinger SC . Mutant p53 drives clonal hematopoiesis through modulating epigenetic pathway. Nat Commun. (2019) 10:5649. doi: 10.1038/s41467-019-13542-2 31827082 PMC6906427

[75] McNerney ME Godley LA Le Beau MM . Therapy-related myeloid neoplasms: when genetics and environment collide. Nat Rev Cancer. (2017) 17:513–27. doi: 10.1038/nrc.2017.60 28835720 PMC5946699

[76] Singhal D Kutyna MM Hahn CN Shah MV Hiwase DK . Therapy-related myeloid neoplasms: complex interactions among cytotoxic therapies, genetic factors, and Aberrant Microenvironment. Blood Cancer Discov. (2024) 5:400–16. doi: 10.1158/2643-3230.BCD-24-0103 39422544 PMC11528189

[77] Chan ICC Zhang P Pan X Castro C Fox N Lewis AM . CDK4/6 inhibition mitigates chemotherapy-induced expansion of TP53-mutant clonal hematopoiesis. Nat Genet. (2026) 58(3):582–92. doi: 10.1038/s41588-026-02526-w 41814002 PMC12987733

[78] Slade D . PARP and PARG inhibitors in cancer treatment. Genes Dev. (2020) 34:360–94. doi: 10.1101/gad.334516.119 32029455 PMC7050487

[79] Todisco E Gigli F Mantiero M Sammassimo S Pastano R Ronchini C . Clinical presentation, diagnosis and management of therapy-related hematological disorders in women with epithelial ovarian cancer treated with chemotherapy and poly-ADP-ribose polymerase inhibitors: A single-center experience. Int J Cancer. (2021) 148:170–7. doi: 10.1002/ijc.33269 32856727

[80] Almanza-Huante E Bataller A Urrutia S Gener-Ricos G Briski RE Kanagal-Shamanna R . Outcomes of patients with therapy-related myeloid neoplasms after treatment with poly(ADP-ribose) polymerase proteins inhibitors for solid tumours. Br J Haematol. (2023) 201:e25–9. doi: 10.1111/bjh.18766 36951293

[81] Marmouset V Decroocq J Garciaz S Etienne G Belhabri A Bertoli S . Therapy-related myeloid neoplasms following PARP inhibitors: real-life experience. Clin Cancer Res Off J Am Assoc Cancer Res. (2022) 28:5211–20. doi: 10.1158/1078-0432.ccr-22-1622 36201165

[82] Martin JE Khalife-Hachem S Grinda T Kfoury M Garciaz S Pasquier F . Therapy-related myeloid neoplasms following treatment with PARP inhibitors: New molecular insights. Ann Oncol Off J Eur Soc Med Oncol. (2021) 32:1046–8. doi: 10.1016/j.annonc.2021.04.015 34107346

[83] Chiusolo P Marchetti C Rossi M Minnella G Salutari V Distefano M . A common pattern of somatic mutations in t-MDS/AML of patients treated with PARP inhibitors for metastatic ovarian cancer. Am J Hematol. (2022) 97:E400–3. doi: 10.1002/ajh.26697 36054600

[84] Kwan TT Oza AM Tinker AV Ray-Coquard I Oaknin A Aghajanian C . Preexisting TP53-variant clonal hematopoiesis and risk of secondary myeloid neoplasms in patients with high-grade ovarian cancer treated with rucaparib. JAMA Oncol. (2021) 7:1772–81. doi: 10.1001/jamaoncol.2021.4664 34647981 PMC8517887

[85] Watson CJ Papula AL Poon GYP Wong WH Young AL Druley TE . The evolutionary dynamics and fitness landscape of clonal hematopoiesis. Science. (2020) 367:1449–54. doi: 10.1126/science.aay9333 32217721

[86] King KY Huang Y Nakada D Goodell MA . Environmental influences on clonal hematopoiesis. Exp Hematol. (2020) 83:66–73. doi: 10.1016/j.exphem.2019.12.005 31893524 PMC7103536

[87] Frick M Chan W Arends CM Hablesreiter R Halik A Heuser M . Role of donor clonal hematopoiesis in allogeneic hematopoietic stem-cell transplantation. J Clin Oncol. (2019) 37:375–85. doi: 10.1200/jco.2018.79.2184 30403573

[88] Wong WH Bhatt S Trinkaus K Pusic I Elliott K Mahajan N . Engraftment of rare, pathogenic donor hematopoietic mutations in unrelated hematopoietic stem cell transplantation. Sci Transl Med. (2020) 12:eaax6249. doi: 10.1126/scitranslmed.aax6249 31941826 PMC7521140

[89] Boettcher S Wilk CM Singer J Beier F Burcklen E Beisel C . Clonal hematopoiesis in donors and long-term survivors of related allogeneic hematopoietic stem cell transplantation. Blood. (2020) 135:1548–59. doi: 10.1182/blood.2019003079 32181816

[90] Awada H Gurnari C Visconte V Durmaz A Kuzmanovic T Tu ZJ . Clonal hematopoiesis-derived therapy-related myeloid neoplasms after autologous hematopoietic cell transplant for lymphoid and non lymphoid disorders. Leukemia. (2024) 38:1266–74. doi: 10.1038/s41375-024-02258-y 38684821 PMC11147764

[91] Rhee JW Chen S Pillai R Boswoth A Oganesyan A Atenzio L . Clonal hematopoiesis is associated with non-myeloid subsequent malignant neoplasms after autologous hematopoietic cell transplantation. Blood. (2024) 144:947–8. doi: 10.1182/blood-2024-200402

[92] Chapman MS Wilks M Boettcher S Mitchell E Dawson K Williams N . Clonal dynamics after allogeneic haematopoietic cell transplantation. Nature. (2024) 635:926–34. doi: 10.1038/s41586-024-08128-y PMC11602715 39478227

[93] Gillis N Abied A Thompson Z Pidala J . Clinical impact of clonal hematopoiesis in hematopoietic cell transplantation: A review, meta-analysis, and call to action. Haematologica. (2024) 109:3952–64. doi: 10.3324/haematol.2024.285392 38899345 PMC11609801

[94] Tanaka T Morita K Loghavi S Wang F Furudate K Sasaki Y . Clonal dynamics and clinical implications of postremission clonal hematopoiesis in acute myeloid leukemia. Blood. (2021) 138:1733–6. doi: 10.1182/blood.2020010483 34115096 PMC8569418

[95] Heuser M Heida B Büttner K Wienecke CP Teich K Funke C . Posttransplantation MRD monitoring in patients with AML by next-generation sequencing using DTA and non-DTA mutations. Blood Adv. (2021) 5:2294–304. doi: 10.1182/bloodadvances.2021004367 33929500 PMC8114555

[96] Xie Y Kazakova V Weeks LD Greber JM Tai J Zhao TY . Effects of donor –engrafted clonal hematopoiesis in allogeneic and autologous stem cell transplantation: A systematic review and meta-analysis. Bone Marrow Transpl. (2024) 59:1585–93. doi: 10.1038/s41409-024-02403-2 39183321 PMC11531373

[97] Avigan ZM Catlett J Bodnar S Pan D Aleman A Sheng T . Clonal hematopoiesis and inflammation predict hematologic toxicity and secondary myeloid malignancies after B-cell maturation antigen-directed chimeric antigen receptor T-cell therapy. Clin Cancer Res. (2025) 31:4333–44. doi: 10.1158/1078-0432.ccr-25-1587 40801897 PMC12521914

[98] Crants SA Olson SS Li Y Bejan CA Bick A Luo L . Radiation therapy and subsequent clonal hematopoiesis: an analysis of a biorepository of 89,782 patients. In: International Journal of Radiation Oncology * Biology * Physics, Poster Q&a Sessions Philadelphia, PA: Elsevier Inc. (2026).

[99] Munzur AD Herberts C Kwan EM Emmett L Sandhu S Buteau JP . Clonal hematopoiesis after 177Lu-PSMA-617 radioligand therapy in prostate cancer. Clin Cancer Res. (2026) 32:2644–52. doi: 10.1158/1078-0432.CCR-25-4001 41649873

[100] Singh A Mencia-Trinchant N Griffiths EA Altahan A Swaminathan M Gupta M . Mutant PPM1D- and TP53-driven hematopoiesis populates the hematopoietic compartment in response to peptide receptor radionuclide therapy. JCO Precis Oncol. (2022) 6:e2100309. doi: 10.1200/PO.21.00309 35025619 PMC8769150

[101] Dutta A Yang Y Le B Zhang Y Abdal-Wahab O Zang C . U2af1 is required for survival and function of hematopoietic stem/progenitor cells. Leukemia. (2021) 35:2382–98. doi: 10.1038/s41375-020-01116-x 33414485 PMC8283943

[102] Chan ICC Wiley BJ Bolton KL . What clonal hematopoiesis can teach us about MDS. Front Oncol. (2022) 12:794021. doi: 10.3389/fonc.2022.794021 35211401 PMC8861516

[103] Coombs CC Zehir A Devlin SM Kishtagari A Syed A Jonsson P . Therapy-related clonal hematopoiesis in patients with non-hematologic cancers is common and associated with adverse clinical outcomes. Cell Stem Cell. (2017) 21:374–382.e4. doi: 10.1016/j.stem.2017.07.010 28803919 PMC5591073

[104] Krönke J Fink EC Hollenbach PW MacBeth KJ Hurst SN Udeshi ND . Lenalidomide induces ubiquitination and degradation of CK1α in del (5q) MDS. Nature. (2015) 523:183–8. doi: 10.1038/nature14610 PMC4853910 26131937

[105] Schneider RK Ademà V Heckl D Järås M Mallo M Lord AM . Role of casein kinase 1A1 in the biology and targeted therapy of del (5q) MDS. Cancer Cell. (2014) 26:509–20. doi: 10.1016/j.ccr.2014.08.001 25242043 PMC4199102

[106] Cheng A Rao Q Liu Y Huang C Li J Huo C . Genomic and expressional dynamics of ovarian cancer cell lines in PARPi treatment revealed mechanisms of acquired resistance. Gynecol Oncol. (2022) 167(3):502–12. doi: 10.1016/j.ygyno.2022.10.011 36270832

[107] Stonestrom AJ Menghrajani KN Devlin SM Franch-Exposito S Ptashkin RN Patel SY . High-risk and silent clonal hematopoietic genotypes in patients with nonhematologic cancer. Blood Adv. (2024) 8:846–56. doi: 10.1182/bloodadvances.2023011262 38147626 PMC10875331

[108] Smith SM Le Beau MM Huo D Karrison T Sobecks RM Anastasi J . Clinical-cytogenetic associations in 306 patients with therapy-related myelodysplasia and myeloid leukemia: the University of Chicago series. Blood. (2003) 102:43–52. doi: 10.1182/blood-2002-11-3343 12623843

[109] Schoch C Kern W Schnittger S Hiddemann W Haferlach T . Karyotype is an independent prognostic parameter in therapy-related acute myeloid leukemia (t-AML): an analysis of 93 patients with t-AML in comparison to 1091 patients with de novo AML. Leukemia. (2004) 18:120–5. doi: 10.1038/sj.leu.2403187 14586477

[110] Wu S Powers S Zhu W Hannun YA . Substantial contribution of extrinsic risk factors to cancer development. Nature. (2016) 529:43–7. doi: 10.1038/nature16166 PMC4836858 26675728

